# Self-Reported Household Impacts of Large-Scale Chemical Contamination of the Public Water Supply, Charleston, West Virginia, USA

**DOI:** 10.1371/journal.pone.0126744

**Published:** 2015-05-07

**Authors:** Charles P. Schade, Nasandra Wright, Rahul Gupta, David A. Latif, Ayan Jha, John Robinson

**Affiliations:** 1 West Virginia Medical Institute (WVMI) & Quality Insights, Charleston, West Virginia, United States of America; 2 Kanawha-Charleston Health Department, Charleston, West Virginia, United States of America; 3 Department of Pharmacy, University of Charleston, Charleston, West Virginia, United States of America; University of Louisville, UNITED STATES

## Abstract

A January 2014 industrial accident contaminated the public water supply of approximately 300,000 homes in and near Charleston, West Virginia (USA) with low levels of a strongly-smelling substance consisting principally of 4-methylcyclohexane methanol (MCHM). The ensuing state of emergency closed schools and businesses. Hundreds of people sought medical care for symptoms they related to the incident. We surveyed 498 households by telephone to assess the episode’s health and economic impact as well as public perception of risk communication by responsible officials. Thirty two percent of households (159/498) reported someone with illness believed to be related to the chemical spill, chiefly dermatological or gastrointestinal symptoms. Respondents experienced more frequent symptoms of psychological distress during and within 30 days of the emergency than 90 days later. Sixty-seven respondent households (13%) had someone miss work because of the crisis, missing a median of 3 days of work. Of 443 households reporting extra expenses due to the crisis, 46% spent less than $100, while 10% spent over $500 (estimated average about $206). More than 80% (401/485) households learned of the spill the same day it occurred. More than 2/3 of households complied fully with “do not use” orders that were issued; only 8% reported drinking water against advice. Household assessments of official communications varied by source, with local officials receiving an average “B” rating, whereas some federal and water company communication received a “D” grade. More than 90% of households obtained safe water from distribution centers or stores during the emergency. We conclude that the spill had major economic impact with substantial numbers of individuals reporting incident-related illnesses and psychological distress. Authorities were successful supplying emergency drinking water, but less so with risk communication.

## Introduction

Chemicals have rarely been associated with reported waterborne disease outbreaks in the US. Such outbreaks were infrequent between 1971 and 2010, and typically affected only a few individuals, as compared with microbe-caused outbreaks, which commonly resulted in hundreds of illnesses. [1–6] The January 2014 chemical contamination of the Elk River in West Virginia (WV) disrupted public water supply to approximately 300,000 homes, closed schools and businesses, and caused hundreds of people to seek medical care for symptoms they associated with water exposure. It is the largest reported outbreak of acute illness related to chemical contamination of water in recent US history.

On January 9, 2014 approximately 10,000 gallons of an industrial chemical used for cleaning coal was discharged less than 2 miles upstream of the water intake serving the municipal water systems of Charleston, WV and the surrounding counties. The chemical, known as Crude MCHM, consisted principally of 4-methylcyclohexanemethanol (MCHM) along with eight other organic chemicals (including propylene glycol phenyl ether and dipropylene glycol phenyl ether). Dispersed into the river, the chemical was rapidly taken into the treatment plant, where it overwhelmed the activated charcoal filtration system and entered the finished water supply. [7,8]

Little information about potential toxicity of MCHM was available. According to Eastman Chemical Company’s safety data sheet, MCHM is harmful if swallowed, causes skin and eye irritation, and at elevated temperatures can cause irritation of the eyes and respiratory tract. [9] No data were available on MCHM’s carcinogenic effects, mutagenic effects, developmental toxicity, specific organ toxicity, bioaccumulation potential, or aspiration risk in humans, although the manufacturer’s proprietary animal testing reportedly showed low potential for mutagenicity and carcinogenicity.[10] The U.S. Centers for Disease Control (CDC) says there should not be any MCHM in drinking water.[11] Initial water testing results on January 10 showed 1.04–3.35 ppm of MCHM at the water intake on the Elk River, and 1.02–1.56 ppm in treated water.[8,11] Over the next four days, MCHM at the water intake declined rapidly to non-detectable levels. [12]

The Governor declared a state of emergency, and the water company issued a "do not use" order within hours, forcing closure of nearly 2,000 water-dependent facilities and 100 schools. This was followed by a federal disaster declaration and a massive joint mitigation action by local, state and federal agencies. [7,8]

From January 13, the water company issued detailed plumbing system flushing guidelines for specific affected areas to allow resumption of drinking water use. Public controversy about the effectiveness of flushing due to residual odor was reinforced by an expert’s subsequent report showing mixed results.[13] The water company lifted all restrictions on January 18. The CDC formally declared the water to be safe for drinking (with a note of caution for pregnant women) only on February 21, 2014.[8]

This incident severely affected the lives and well-being of thousands of WV residents, many of whom complained of lingering odor from the water supply, despite flushing. The health authorities attempted to clarify that the persisting odor was unrelated to detrimental health effects, but their statements were often perceived to be inconsistent; public mistrust on water quality remained.[8,14]

The Kanawha-Charleston Health Department (KCHD), whose jurisdiction encompasses 200,000 individuals in the most-affected area, surveyed residents to assess the health and economic impact of the water crisis and identify issues requiring further intervention. The survey also aimed to understand public perception of risk communication by federal, state, and local health agencies and the water company. The objectives of this study were to describe: (1) physical and psychological symptoms that occurred in the affected population, (2) self-reported direct economic costs, (3) personal and household protective behavior, and (4) perceptions of quality of official communication.

## Methods

We conducted a cross-sectional, population-based telephone survey from April 3–8, 2014 in Kanawha County, West Virginia, approximately 90 days after the chemical spill incident. The Institutional Review Board of the University of Charleston approved the current research as exempt. We obtained verbal informed consent from participants.

### Survey instrument

We developed a 75 item questionnaire about household experience during and after the water emergency (S1 Text: Survey questionnaire and codebook). Questions included demographics (age, race, gender, education, employment status, occupation, and income); usual water source; how and when the respondent learned of the water emergency; use of water during and after the emergency; sources of water during the emergency; assessment of communication received during the emergency, by source (federal, state, local, and water company) and domain (helpfulness, understandability, trustworthiness); symptoms of illness related to the water and sources of care and advice; economic impact of the crisis; psychological distress during and after the incident; use of water after the incident; and perceived safety of the water supply. Where possible, we used validated items from the Behavioral Risk Factor Surveillance System (BRFSS),[15] e.g., demographic questions. We developed most questions de novo, as they were specific to the water spill situation.

We pre-tested the survey instrument by face-to-face interview of a group of 5 community opinion leaders. This resulted in changes to the communication rating scale (from Likert to A-F) and substitution of the four locally-developed psychological distress questions for the BRFSS items, which the pre-testers believed to be too focused on depression symptoms. We trained 27 health department staff and 28 volunteers to administer the survey.

### Study sample

We obtained three lists of random telephone numbers in Kanawha County, consisting of almost 4,000 land line numbers and 2,000 cellular phone numbers. The eligible study population was defined as residents of Kanawha County, living at the current address on January 9, 2014, and having the West Virginia American Water Company as their source of residential water supply.

The interviewers made phone calls from the three lists of random telephone numbers, eliminating disconnected/ non-working numbers, fax machines, and duplicate numbers; a total of 1,246 calls were completed. After we excluded 390 ineligible households, 498 eligible respondents voluntarily completed the interview, and were included in the final analysis, for a response rate of 59.8% (498/(1,246–390)). Please refer to Survey Administration (S2 Text: Details of survey administration) for more details.

### Statistical Analysis

We used SAS (Statistical Analysis System) software v. 9.3 (SAS Institute Inc., Cary, NC, USA) for statistical analyses.

We performed detailed univariate descriptive analysis, using frequency distributions for categorical data, and means for continuous measures such as age and household size. We estimated individual illness rates from household illness rates and household size by fitting a binomial model of illness rates by household size, assuming individuals became sick independently of other household members.

We tabulated respondent assessments of 4 different sources of public information during the emergency: federal, state, local and water company. Respondents rated each source on a scale of A-F in three domains: clarity and understandability; helpfulness; and trustworthiness, yielding 12 items (4 sources x 3 domains). We converted letter grades to numbers (‘A’ = 1…’F’ = 6) to calculate means and correlations across sources and domains.

Because of correlations among the psychological distress items, we computed a composite measure with a range of 0 to 12 (high = greater distress) by summing the individual item scores and subtracting from 16. We used the median of this composite measure to define individuals with higher and lower levels of distress, used in bivariate analysis of psychological distress among subgroups of the surveyed population.

We estimated overall household costs due to the incident by adjusting the distribution of categorical self-reported household expenditure levels by household size to the Kanawha County 2010 US Census household composition. We fit the adjusted categorical expense frequencies to a log normal distribution, whose mean was the estimated mean household expenditures in Kanawha County, and extrapolated to the county population.

We compared our findings for consistency with two concurrent studies performed by the WV Bureau for Public Health: surveillance of number of individuals receiving hospital emergency department services for illnesses they thought related to the water,[16] and a small geographically-based household survey with similar questions to ours. [17]

## Results

The final analysis includes 498 Kanawha County households, each represented by an eligible adult respondent. Interviewers contacted 61 of them (12%) through cell phones, and the remainder through landlines.

### Respondent and Household Characteristics

The basic demographics of the surveyed population are shown in S1 Table: Age and gender distribution of the respondents. Of the 488 respondents who gave their age, 214 (44%) were 65 years or older; the mean respondent age was 61; of 483 respondents whose age and gender were reported, 332 (67%) were female. Nearly all respondents were white; most had at least completed high school and were either employed or retired. Sixty-five percent (272) of the households reported annual income of $50,000 or less. In 8 households (2%) the respondent said someone had been pregnant during the water emergency. Twelve reported having at least one member younger than age 1.

In comparison, of the 2010 U.S. Census Kanawha County population aged 18 or older, 53% were female and 21% were aged 65 and older.[18] The average household had 2.4 persons, as compared with 2.3 in the Census.

### Personal and household protective behavior

Forty eight percent (240) of respondents said their household used purchased or bottled water during the emergency. However, of those who said their household had any bottled water at the time of the spill, 83 (35%) said they had less than one gallon on hand then. Fewer than 30% said they had more than two gallons. Sixty eight percent of all households surveyed had less than one gallon of bottled water at the time of chemical spill; 52% had none (Table 1).

**Table 1:** Bottled water on hand at time of emergency.

Amount of water on hand	Number of respondents	Percentage of respondents with bottled water	Percentage of all respondents
**<1 Gallon**	83	34.60%	16.70%
**1–2 Gallons**	71	29.60%	14.30%
**3–5 Gallons**	45	18.80%	9.00%
**> 5 Gallons**	28	11.70%	5.60%
**Don't know**	13	5.40%	2.60%
**Households with bottled water**	240	-	48.20%
**Households without bottled water**	258	-	51.80%
**Total households**	498	-	-

Table 2 shows that 23% of respondents (115/498) said their household used water during the emergency in ways contrary to official advice (sanitation and fire control only). In 43 households (8%), someone drank water during the emergency; in 90 (18%), someone bathed or showered. Households with younger respondents reported more water use during the emergency than those with older respondents (36% of those aged 18–44 vs. 19% of those older than 65, p = 0.0052). Male respondents reported more unapproved household water use than females (30% vs, 19%, p = 0.0078). Respondent education, employment status, and income were not significantly associated with reported household water use.

**Table 2:** Households using water for purposes other than sanitation and fire fighting during the emergency.

Type of water use (after "Do not use" order)	Number of households	Percentage of households with any unapproved use	Percentage of all households
**Drank**	43	37.4%	8.6%
**Washed hands**	64	55.7%	12.9%
**Brushed teeth**	47	40.9%	9.4%
**Cooked**	34	29.6%	6.8%
**Washed clothes**	51	44.3%	10.2%
**Bathed/showered**	90	78.3%	18.1%
**Dishwashing**	49	42.6%	9.8%
**Gave to pets**	32	27.8%	6.4%
**Watered plants**	27	23.5%	5.4%
**Any unapproved use**	115	-	23.1%
**Only for sanitation/fire control**	382	-	-
**Total respondents (includes 1 "don't know")**	498	-	-

Ninety two percent of households (457) sought other water sources during the emergency (Table 3). Of these, 284 households (57%) said they used water distribution centers in their own city or elsewhere. Three hundred fifty three of the households (77%) purchased water at large stores or grocery stores. Households using water distribution centers usually learned about them from television (159; 56%) or word of mouth (84; 30%). Fewer learned about them from radio, social media, or other sources.

**Table 3:** Water sources used by households during emergency.

Source of Water	Number of households	Percentage of households seeking other sources	Percentage of all households
**Distribution center in town of residence**	251	54.9%	50.4%
**Distribution center elsewhere**	98	21.4%	19.7%
**Purchased from large store or grocery**	353	77.2%	70.9%
**Purchased from gas station or convenience store**	74	16.2%	14.9%
**Well water**	14	3.1%	2.8%
**Friend**	104	22.8%	20.9%
**Rainwater**	47	10.3%	9.4%
**Some other source**	24	5.3%	4.8%
**Tried to get water**	457	-	91.8%
**Did not try to get water**	40	-	-
**Total respondents (includes 1 "don't know")**	498	-	-

Of the 457 households who had sought other water sources, 57 (12%) reported that someone went without safe drinking water for one or more days. Among those households, common reasons offered for not having safe water included exhaustion of supplies at stores or distribution centers, transportation, cost, and problems finding distribution centers. However, the most frequently cited reason, stores without water, affected 22 households or fewer than 5%.

### Assessment of official communication during emergency

Scores of ‘A,’ ‘C,’ and ‘F’ predominated in responses to the 12 communication items. The scores were highly correlated with one another (p<0.0001). Differing across the items was the proportion of respondents rating each one ‘A’ as compared with those rating it ‘F.’ This difference is illustrated in Table 4, which shows that many respondents had a negative bias towards information from Federal authorities and the water company, a more neutral position on WV state communications, and a more positive view of information from local officials. It also indicates that the average respondent gave a grade of ‘C’ or worse (3.0 or greater) to most sources and domains.

**Table 4:** Communication quality scores: differences in numbers of respondents rating each domain of communication A and those rating it F on each of three categories.

Domains of risk communication	Communication rating item (categories)	Difference between numbers (of respondents) rating 'A' and 'F' (Count)	Difference between numbers (of respondents) rating 'A' and 'F' (Percentage)	Mean Item Grade
**Federal Information**	clear and understandable	-57	-12.6	3.6
	helpful	-69	-15	3.7
	trustworthy	-112	-24.6	4.0
**State (WV) Information**	clear and understandable	-4	-0.8	3.1
	helpful	-12	-2.5	3.2
	trustworthy	-58	-12.3	3.6
**WV American Water company Information**	clear and understandable	-66	-13.9	3.6
	helpful	-65	-13.6	3.6
	trustworthy	-115	-24.2	3.9
**Local Information**	clear and understandable	76	17	2.7
	helpful	69	15.3	2.7
	trustworthy	39	8.7	2.9

Positive difference = respondents viewed information favorably

Negative difference = respondents did not view information favorably.

### Household economic impact

One hundred thirty four of the households (29% of those seeking other water sources) traveled out of area to get water. Of those, 62 (46%) purchased water; 63 (47%) got water from friends or relatives. Fifty three households (11%) stayed overnight, and 40% of them paid to do so. Sixty seven respondent households (13%) had one or more members miss work because of the crisis, missing a median of 3 days of work. In 34 households, someone owned a business; 8 of those businesses were ordered to close because of the emergency. In 78 (15%) of the households, someone was told not to come in to work because of the emergency. Only 31 (40%) of these received pay for missed work. Of the 443 households reporting extra expenses due to the crisis, 203 (46%) said they spent less than $100, but 43 (10%) reported expenses of more than $500 (Table 5). Based on self-reports, we estimated mean household expenses in the surveyed population of $245; weighting the surveyed population to reflect the household composition of Kanawha County reduced estimated mean expenditures to $206.

**Table 5:** Extra expenses incurred by households during emergency.

Estimated amount spent	Households		
	Number	Percent of those who knew expenditures	Adjusted to 2010 household composition
**<$100**	203	45.8%	50.7%
**$100-$250**	114	25.7%	25.7%
**$250-$500**	83	18.7%	16.2%
**$500+**	43	9.7%	7.4%
**Don't know**	55	-	-
**Total**	498	-	-
**Mean household expenditure**	-	$245	$206

Total household expenditures were consistent with household efforts to avoid or mitigate the effects of the spill. Thus, travel and travel intensity (whether a household stayed overnight out of the area or paid to stay overnight) was positively associated with total expense reported (p<0.0001). Of households paying to stay overnight (one or more nights), 52% incurred more than $500 in expenses, while a similar proportion of households who did not travel spent less than $100. Loss of work due to the emergency was associated with higher reported expenses (p<0.0001). The strongest association was with someone sick in the household: 83% of households without anyone sick said they spent less than $100, whereas 60% of households with illness spent more than $500 (p<0.0001).

### Reported health effects

Thirty two percent of households (159/498) reported someone with illness believed to be related to the chemical spill. Based on the assumption that individual illnesses occurred independently of one another within households, we estimated an individual illness rate of at least 16.3% in the surveyed population. (S1 Fig: Actual and estimated physical illness rates by household size). Of the households with someone ill, 101 (64%) reported rash or skin irritation as a symptom. Other commonly-reported symptoms included nausea or vomiting (42, 26%), diarrhea or abdominal cramps (43, 27%), headache or dizziness (40, 25%), or eye irritation (42, 26%).

Most symptoms occurred after the “Do not use” order was in effect (71/159 households, 45%), but 25% occurred before and 38% during that time. In 40 (25%) of the households with someone sick, individual(s) sought medical treatment or advice, most commonly from a primary care physician (18, 45%), urgent care center (10, 25%), or hospital emergency department (11, 28%). Respondents in three households reported someone admitted to the hospital. Eighty three respondents (52% of households where someone was sick) said that at least one person with symptoms did not seek medical attention or advice. Of these (56, 67%) said they weren’t sick enough.

Households reporting water use during the emergency were not significantly more likely to report having someone sick (36% vs 31%, p = 0.3233). Symptoms beginning before or after the emergency were not associated with water use during the emergency.

### Psychological distress

Respondents experienced more frequent symptoms of psychological distress during and within 30 days after the emergency than 90 days later. For example, 37% of respondents indicated they were worried “all the time” during the emergency, whereas only 17% were 90 days later. Meanwhile the proportion not feeling worried at all increased from 24% to 44%. The individual symptoms were highly correlated (p<0.0001); S2 Fig: Level of psychological distress during and after emergency, percent of respondents worried, S3 Fig: Level of psychological distress during and after emergency, percent of respondents stressed, S4 Fig: Level of psychological distress during and after emergency, percent of respondents angry, and S5 Fig: Level of psychological distress during and after emergency, percent of respondents depressed. The composite measure of psychological distress had a mean and median of 5, and was unimodal, but not normally distributed. Table 6 shows the relationship between psychological distress and selected demographics, presenting the number and proportion of respondents in each group experiencing more or less psychological distress than the median. Females, younger respondents, and respondents in larger households experienced more distress than males, older respondents, and respondents in smaller households, respectively (p<0.0001). There was no significant association with household income.

**Table 6:** Demographics and psychological distress.

	Respondent distress during episode			
	< = Median		>Median	
	N	Percent	N	Percent
Respondent age (p<0.0001)				
18–44	26	12	51	21
45–64	64	29	127	51
65+	134	60	70	28
Respondent gender (p<0.0001)				
Male	94	42	60	24
Female	131	58	191	76
Household income (p = 0.2408)				
<$25,000	52	23	78	31
$25–50,000	64	28	66	26
$50–75,000	48	21	38	15
>$75,000	28	12	30	12
Don't know	12	5	9	4
Refused	24	11	32	13
Household size (p<0.0001)				
1	69	30	50	20
2	105	46	96	38
3 or more	54	24	107	42

### Post-event water consumption behavior

Four hundred thirty six respondents (88%), interviewed after the spill, said that they had believed the water was safe prior to the incident; after the spill, only 143 (29%) did. Most households (467/498) reported using water company-supplied water 90 days after the event, but 6% of the population was still not using the water. However, only 169 households reported that they were actually drinking the water. Sixty six percent of households (328/467) were not drinking the water three months out. Most respondents of households with some water use said their households were using water for hand washing (86%), dish washing (86%), clothes washing (96%), and bathing (96%). In 30 (18%) of the households where people were drinking the water, one or more persons was not.

### Consistency with other concurrent sources

S3 Text: Comparison with the CDC/ WV DHHR CASPER survey compares our findings with CDC's CASPER survey on age and sex composition of households, and water sources during emergency. KCHD households included more older individuals and fewer working age adults than the CASPER survey. The vast majority of respondent households in both surveys tried to get other water sources besides the public water supply during the emergency. Similar proportions used water from all sources except for friends and relatives, which households in the CASPER survey used twice as often (CASPER, 44% vs. KCHD, 23%). With respect to other major areas of inquiry, including date of first awareness, source of information, availability of bottled water, traveling to get safe water, and symptom frequencies with illness, the two surveys produced largely consistent results, where questions were similar.

According to the Bureau for Public Health, 369 people went to hospital emergency departments with symptoms related to the water between January 9 and January 23, 2014.[19] In the KCHD survey, 12 respondents said someone went to the emergency room or was admitted to the hospital due to symptoms related to the water. Extrapolating to the entire county population implies about 4,600 ER visits countywide, more than ten times the rate from active surveillance.

## Discussion

In a large study of groundwater used for drinking, Toccalino and colleagues [20] found low-level chemical contamination to be quite common in the United States. The most frequently detected organic chemicals were herbicides, disinfection byproducts, and solvents; while trace elements, radon, and nitrate were the most common inorganic chemicals. The principal chemical involved in the present study (MCHM) has not been previously reported as a drinking water contaminant.

### Health impact

Literature on mass exposures to larger quantities of chemical contaminants in water supplies released at a single point in time is scarce. Rowland and co-workers [21] reported on the immediate consequences of a spill of aluminum sulfate into a water supply in North Cornwall, England. This study showed that a group of survey respondents who were exposed to contaminated water were more likely to complain of eighteen symptoms than control subjects from another area. However, the researchers could not exclude anxiety and publicity as possible causes of the differences in symptom reporting. In the current survey, most of the perceived physical illness related to use of contaminated water were mild and non-specific dermatological (skin rashes/ irritation) and gastrointestinal symptoms.

Only a minority required medical attention in this episode. The lack of major clinical findings in the surveillance study suggests a low likelihood of short-term physical harm following exposure to the crude MCHM in water. However the large number of individuals with symptoms does raise the question of how to handle the situation more effectively in future incidents, e.g., offering guidance about when seeking medical care might be beneficial. Long term consequences may be different. They will be difficult to quantify because few people consumed the water during the crisis and concentrations were modest An adult drinking 2 liters of water per day throughout the crisis probably received a total dose of MCHM less than 1 mg/kg body weight. From these data we cannot assess the extent to which emotional distress associated with loss of water and lack of reassurance from authorities augmented perceived physical illness.

The North Cornwall chemical spill incident led to further publications, with conflicting results, just as this episode has already produced studies with different findings. Owen and Miles found that hospital discharge rates in the affected area were increased during the five years following the spill, with no specific condition predominant.[22] These investigators later reported that mortality in the affected region had not increased compared with an unexposed population.[23] McMillan’s team found no significant differences in psychological testing of children exposed to the contaminant compared with unexposed children, [24] whereas Altman reported signs of cerebral dysfunction in adults who had been exposed. [25] Golding and co-workers studied outcomes of pregnancy in women exposed to the water and failed to find significant differences compared with control groups, although the small number of pregnancies was a limitation.[26] As in the present situation, official reports weren’t uniformly accepted as reassurance.[27,28]

Following an episode of mass contamination of water with organic chemicals, Fowle and colleagues [29] conducted population surveys, finding higher levels of symptoms in those exposed compared with unexposed persons. They noted that symptoms were correlated with noticing the chemicals’ taste and odor, which is particularly relevant to incident we described. Drinking the water, but not other contact with it, was associated with symptoms. In that episode, public officials advised people not to consume the water, to which most of the population complied, similar to our findings.

The discrepancy between the surveillance-reported and survey-estimated numbers of ER visits is striking. Several possible explanations may apply. Some of the KCHD cases may have gone to the ER for other causes, and the respondent may have incorrectly recalled the reason. Some of the people who went to the ER may not have complained of water-related symptoms, or they may not have been recorded as such. The time window of the KCHD survey was open ended, and no specific hospitals were identified, whereas the surveillance was for a short period of time in selected hospitals. While the relatively benign illnesses reported in surveillance are reassuring, both sources paint a picture of considerable short term morbidity and attendant medical expense absorbed by individuals, their insurers, and the community, buttressed by the significant association of symptoms with reported household expense.

### Risk communication

The CDC views emergency/ risk communication as the critical process of delivering the best possible science-based advice to all concerned, "with nearly impossible time constraints, and ultimately, to accept the imperfect nature of the choices as the situation evolves".[30] The six cardinal principals are laid down as: be first, be right, be credible, express empathy, promote action, and show respect. Our analysis of the public perception of the risk communication from 4 sources showed that the public perception of communication received from federal agencies and the water company was more negative in all 3 domains. The state-level information fared better compared to these two, but the most positive public perception was of local agencies. Credibility suffered when advice from authorities seemed inconsistent; delayed guidance for pregnant women did not enhance public trust.

Rundblad reported somewhat similar results, finding that the public was more receptive to receiving risk communication through local radio over postal information from the water company following routine drinking water incidents in UK. [31] Studying the May 2010 Boston water crisis, researchers noted that crisis communication remains one of the major determinants in minimizing unfavorable public perceptions.[32] Our results suggested that risk communication in this episode was not as good as it might have been, in contrast to the generally successful Boston effort. [33]

### Economic Impact

Given Kanawha County’s estimated 82,765 households,[34] these results suggest total direct household expenses approximately $17 million. As the company responsible for the spill declared bankruptcy days afterward, it is unlikely that residents will see appreciable compensation for their losses aside from the approximately $100,000 raised and distributed by United Way to assist those who had lost wages. [35]

### Limitations

Because of its sample, the current survey is unlikely to represent the population of Kanawha County, but may be reasonably representative of households, judging from the similarity of results with the CASPER survey. Moreover, adjustment of results to the age/sex distribution of the county population changed frequencies only slightly. Responses by cell phone differed significantly from those by landline (smaller household size, younger age, more current employment), but only a minority of respondents used cell phones. We do not know the current proportion of households using cell phones, landlines, or both in Kanawha County.

We did not assess symptom prevalence against a control group outside the affected area. However, prevalence of the more common symptoms was much higher than could reasonably be expected in a healthy population. We did not obtain health histories of household members, some of whom might have had chronic conditions that caused symptoms they believed were related to the water. However, prevalence of chronic diseases increases with age, whereas household illness rates in our survey declined as the number of persons age 65 or older in the household increased (p<0.0001).

Discrepancies in responses to similar questions limit the accuracy of conclusions. For example, in 6% of the surveys, the household count derived from gender-specific counts differed from that calculated from age-specific counts, usually by 1. The number of households with someone ill who did not seek medical advice should have been greater than or equal to the difference between total households with illness and the number where at least one person sought care. In fact, it was substantially less.

The questions on psychological distress were not previously validated, and asking for both current and during event data in the same interview may have introduced bias.

Recall bias is potentially significant in a survey 90 days after the event. Many of the more important items, such as whether or not a respondent sought medical care, are unlikely to be forgotten. Prestige bias may have affected results, as interviewers identified themselves with the health department, possibly inflating positive responses local source communication items. Although recency and primacy could have affected responses to longer multiple choice questions, in many cases the interviewer only read the stem and did not prompt with specific choices. Response frequencies for most of the other items show little clustering on the first or last choice.

The grade levels for communication items may have been confusing. Frequency distributions showed little central tendency, owing in part to an apparent aversion to assigning a grade of ‘E’.

Economic data were self-reported. Although they are largely consistent, we do not have objective data such as receipts, to validate individual responses.

Finally, the associations observed in this cross-sectional study cannot be interpreted as demonstrating causation. Additional studies, especially clinical studies in occupationally-exposed humans, would be needed to assess acute health effects of MCHM exposure; careful and repeated measures of actual exposure individual exposure, which we could not obtain, would have been necessary to determine chronic effects.

## Conclusions

The water crisis of January 2014 had a significant economic impact on households in Kanawha County. At least 16% of the surveyed population had symptoms they believed were caused by the contaminated water. The incident affected psychological well-being and resulted in widespread distrust of the public water system that had not resolved 90 days after the event. Mitigation efforts varied in effectiveness, with water distribution reaching the majority of households leaving few people without access to safe water, while risk communication efforts were less successful. As in other reported water disasters, compliance with restrictions on water use was high.

## Supporting Information

S1 Text Survey questionnaire and codebook. (DOCX)

S2 Text Details of survey administration. (DOCX)

S3 Text Comparison with the CDC/ WV DHHR CASPER survey. (DOCX)

S1 Table Age and gender distribution of the respondents. (DOCX)

S1 Fig Actual and estimated physical illness rates by household size. (DOCX)

S2 Fig Level of psychological distress during and after emergency, percent of respondents worried. (DOCX)

S3 Fig Level of psychological distress during and after emergency, percent of respondents stressed. (DOCX)

S4 Fig Level of psychological distress during and after emergency, percent of respondents angry. (DOCX)

S5 Fig Level of psychological distress during and after emergency, percent of respondents depressed. (DOCX)

## References

[1] Centers for Disease Control and Prevention (2013) Surveillance for waterborne disease outbreaks associated with drinking water and other nonrecreational water—United States, 2009–2010. MMWR Morb Mortal Wkly Rep 62: 714–720. 24005226 PMC4585624

[2] Barwick RS, Levy DA, Craun GF, Beach MJ, Calderon RL (2000) Surveillance for waterborne-disease outbreaks—United States, 1997–1998. MMWR CDC Surveill Summ 49: 1–21. 10843502

[3] Kramer MH, Herwaldt BL, Craun GF, Calderon RL, Juranek DD (1996) Surveillance for waterborne-disease outbreaks—United States, 1993–1994. MMWR CDC Surveill Summ 45: 1–33. 8600346

[4] Lee SH, Levy DA, Craun GF, Beach MJ, Calderon RL (2002) Surveillance for waterborne-disease outbreaks—United States, 1999–2000. MMWR Surveill Summ 51: 1–47. 12489843

[5] Levy DA, Bens MS, Craun GF, Calderon RL, Herwaldt BL (1998) Surveillance for waterborne-disease outbreaks—United States, 1995–1996. MMWR CDC Surveill Summ 47: 1–34. 9859954

[6] Moore AC, Herwaldt BL, Craun GF, Calderon RL, Highsmith AK, Juranek DD (1993) Surveillance for waterborne disease outbreaks—United States, 1991–1992. MMWR CDC Surveill Summ 42: 1–22. 8232179

[7] Huffington Post (2014) West Virginia Water Crisis. Available: http://www.huffingtonpost.com/tag/west-virginia-water-crisis/. Accessed 11/25/2014.

[8] Charleston Daily Mail (2014) Two months after spill, residents weigh whether to trust water Available: http://www.charlestondailymail.com/News/201403120098. Accessed 11/25/2014.

[9] (2011) Safety Data Sheet Crude MCHM. Available: http://ws.eastman.com/ProductCatalogApps/PageControllers/MSDS_PC.aspx?Product=71014291. Accessed 12/13/2014.

[10] (2014) Questions and Answers Regarding Eastman’s Assistance in the Emergency Response to the Spill of Crude MCHM in Charleston, West Virginia. Available: http://www.eastman.com/literature_center/misc/Q_and_A_West_Virginia_Spill.pdf. Accessed 12/13/2014.

[11] Centers for Disease Control and Prevention (2014) 2014 West Virginia Chemical Release. Available: http://www.bt.cdc.gov/chemical/MCHM/westvirginia2014/. Accessed 11/25/2014.

[12] (2014) WVAW Operation Log As Of 18 Jan At 1800. Available: http://www.dhsem.wv.gov/Documents/Sampling%20Results/OPERATION%20LOG%2018JAN1800%20Water.pdf. Accessed 12/13/2014.

[13] Adams C, Whelton A, Rosen J (2014) West Virginia Testing Assessment Project (WV TAP) Literature Review. Available: http://www.dhsem.wv.gov/WVTAP/test-results/Documents/POSTED_WVTAP%20Health%20Effects%20Lit%20Review%20v1.5%20031714.pdf. Accessed 12/13/2014.

[14] White K (2014) More questions than answers about water at town hall meeting. Available: http://www.wvgazette.com/News/201401270198#sthash.hvUokASh.dpuf. Accessed 12/14/2014.

[15] Centers for Disease Control and Prevention (2014) Behavioral Risk Factor Surveillance System: BRFSS 2013 Survey Data and Documentation Available: http://www.cdc.gov/brfss/annual_data/annual_2013.html. Accessed 2/5/2015.

[16] West Virginia Department of Health and Human Resources (2014) Elk River Chemical Spill Medical Data. Available: http://www.dhhr.wv.gov/News/chemical-spill/Documents/PRFindings.pdf. Accessed 12/2/2014.

[17] Centers for Disease Control and Prevention (2014) Disaster Response and Recovery Needs of Communities Affected by the Elk River Chemical Spill, West Virginia. Available: http://www.dhhr.wv.gov/News/2014/Documents/WVCASPERReport.pdf.

[18] U.S. Bureau of the Census (2011) 2010 Census Demographic Profiles—West Virginia. Available: http://www2.census.gov/census_2010/03-Demographic_Profile/West_Virginia/. Accessed 4/28/2014.

[19] West Virginia Department of Health and Human Resources (2014) Elk River Chemical Spill Health Effects Findings of Emergency Department Record Review. Available: http://www.wvdhhr.org/Elk%20River%20Chemical%20Spill%20Health%20Effects%20-%20Findings%20of%20Emergency%20Department%20Record%20Review.pdf. Accessed 12/2/2014.

[20] Toccalino PL, Norman JE, Scott JC (2012) Chemical mixtures in untreated water from public-supply wells in the U.S.—occurrence, composition, and potential toxicity. Sci Total Environ 431: 262–270. 10.1016/j.scitotenv.2012.05.044 22687436

[21] Rowland A, Grainger R, Smith RS, Hicks N, Hughes A (1990) Water contamination in North Cornwall: a retrospective cohort study into the acute and short-term effects of the aluminum sulphate incident in July 1988. J R Soc Health 110: 166–172. 2123251 10.1177/146642409011000507

[22] Owen PJ, Miles DP (1995) A review of hospital discharge rates in a population around Camelford in North Cornwall up to the fifth anniversary of an episode of aluminium sulphate absorption. J Public Health Med 17: 200–204. 7576804 10.1093/oxfordjournals.pubmed.a043093

[23] Owen PJ, Miles DP, Draper GJ, Vincent TJ (2002) Retrospective study of mortality after a water pollution incident at Lowermoor in north Cornwall. BMJ 324: 1189 12016184 10.1136/bmj.324.7347.1189 PMC111112

[24] McMillan TM, Dunn G, Colwill SJ (1993) Psychological testing on schoolchildren before and after pollution of drinking water in North Cornwall. J Child Psychol Psychiatry 34: 1449–1459. 8294530 10.1111/j.1469-7610.1993.tb02102.x

[25] Altmann P, Cunningham J, Dhanesha U, Ballard M, Thompson J, Marsh F. (1999) Disturbance of cerebral function in people exposed to drinking water contaminated with aluminium sulphate: retrospective study of the Camelford water incident. BMJ 319: 807–811. 10496822 10.1136/bmj.319.7213.807 PMC314205

[26] Golding J, Rowland A, Greenwood R, Lunt P (1991) Aluminium sulphate in water in north Cornwall and outcome of pregnancy. BMJ 302: 1175–1177. 2043811 10.1136/bmj.302.6786.1175 PMC1669867

[27] Coggon D (1991) Camelford revisited. BMJ 303: 1280–1281. 1747668 10.1136/bmj.303.6813.1280 PMC1671417

[28] Lawson R (1991) Camelford revisited. BMJ 303: 1480 1773167 10.1136/bmj.303.6815.1480-b PMC1671687

[29] Fowle SE, Constantine CE, Fone D, McCloskey B (1996) An epidemiological study after a water contamination incident near Worcester, England in April 1994. J Epidemiol Community Health 50: 18–23. 8762348 10.1136/jech.50.1.18 PMC1060198

[30] Centers for Disease Control and Prevention (2012) Crisis & Emergency Risk Communications Manual. Available: http://emergency.cdc.gov/cerc/resources/pdf/cerc_2012edition.pdf. Accessed 11/25/2014.

[31] Rundblad G, Knapton O, Hunter PR (2014) The Causes and Circumstances of Drinking Water Incidents Impact Consumer Behaviour: Comparison of a Routine versus a Natural Disaster Incident. Int J Environ Res Public Health 11: 11915–11930. 10.3390/ijerph111111915 25411725 PMC4245651

[32] Galarce EM, Viswanath K (2012) Crisis communication: an inequalities perspective on the 2010 Boston water crisis. Disaster Med Public Health Prep 6: 349–356. 10.1001/dmp.2012.62 23241465

[33] Kahn JP, Aucoin D (2010) Long-honed alert system passes its test run. Available: http://www.boston.com/news/local/massachusetts/articles/2010/05/05/as_test_run_for_disaster_alert_system_passed/?page=1. Accessed 12/5/2014.

[34] U.S. Bureau of the Census (2014) State and County Quick Facts, Kanawha County, WV Available: http://quickfacts.census.gov/qfd/states/54/54039.html. Accessed 12/14/2014.

[35] (2014) United Way and Partner Agencies Continue WV Emergency Fund Distribution. Available: http://wvadvocates.org/news/2014/02/07/united-way-and-partners-continue-wv-emergency-fund-distribution/. Accessed 12/6/2014.

