# Correction: Self-Reported Household Impacts of Large-Scale Chemical Contamination of the Public Water Supply, Charleston, West Virginia, USA

**DOI:** 10.1371/journal.pone.0131143

**Published:** 2015-06-16

**Authors:** Charles P. Schade, Nasandra Wright, Rahul Gupta, David A. Latif, Ayan Jha, John Robinson

There is an error in reference 13. The correct reference is: Whelton AJ, Rosen JS, Clancy J, Clancy T, Ergul A (2014) The Crude MCHM Chemical Spill 10‐Home Study: Tap Water Chemical Analysis. Available: http://www.dhsem.wv.gov/WVTAP/testresults/Documents/POSTED%2010%20Home%20Study%20Chemical%20Analysis%20Report_FINAL.pdf. Accessed 5/21/2015.
