# Supplementary material for: Self-Reported Household Impacts of Large-Scale Chemical Contamination of the Public Water Supply, Charleston, West Virginia, USA
Source: PLoS One. 2015 May 7;10(5):e0126744. doi: 10.1371/journal.pone.0126744 (PMC4423935; doi:10.1371/journal.pone.0126744)
Supplement: S1 Text — (DOCX) [file pone.0126744.s001.docx]

**Community Assessment**

*
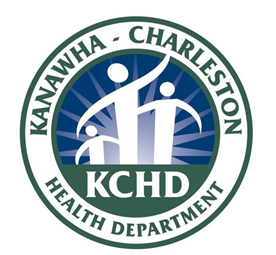
*

**Public Health Emergency Response**

**Elk River Chemical Spill**

**January 9th, 2014**

*Telephone number ________________*

*Date of call ____________________*

*Interviewer _____________________*

*Survey number ___________________*

Hello. I am calling from the Kanawha Charleston Health Department to ask how the chemical spill and water crisis affected you.

Q1. Would you be willing to answer a few questions to help us?

[ ] Yes =1

[ ] No =2

**If No:** Thank you for speaking with me. Good bye. **EXIT**

**If Yes:** The survey will take about 20 minutes. Is now a good time to talk?

[ ] Yes

[ ] No

**If Yes: GOTO INTRODUCTION**

**If No:** *Ask for a better time, and negotiate to set a time during the available calling times. Write the callback time here:*

[ ] Call back at ______ on _____

(time) (day)

[ ] Could not agree on time

**If could not agree:** Thank you for speaking with me. I’m sorry we couldn’t set a convenient time. Good bye. **EXIT**

**INTRODUCTION**

The Kanawha Charleston Health Department is sponsoring this survey. Your responses are confidential. You are not required to answer any question that makes you uncomfortable, and you may stop answering questions at any time. Your decision to answer or not to answer these questions will not affect services you receive from the health department. If you have questions about the survey, or want more information, you may call (304)348-6494.

We’ll start with a few questions to be sure you were in the area where the chemical spill got into the water.

Q2. Did you live at your current address on January 9, 2014

[ ] Yes =1

[ ] No =2

**If No:** Thank you very much, but we are only talking with people who lived at the same address since January 9. Good bye. **EXIT.**

Q3. Before January 9, 2014, what was the source of water used for drinking, cooking, and bathing at your residence? Did it come from:

*Read list:*

[ ] The West Virginia American Water Company =1

[ ] Some other water company =2

[ ] A well or another private source =3

[ ] Don’t know =9

**If WVAWC (1): GOTO BASIC INFORMATION**

**If well or some other:** Thank you for participating. At this time we are only interviewing people who got their water from the West Virginia American Water Company. Good bye. **EXIT**

**If don’t know (9):**

Q4. Did your household receive an automated telephone call (“Robocall”) or text message on January 9 or January 10 telling you not to use your water?

[ ] Yes =1

[ ] No =2

[ ] Don’t know =9

**If no or don’t know**: Thank you for participating. At this time we are only interviewing people who got their water from the West Virginia American Water Company. Good bye. **EXIT**

**BASIC INFORMATION**

Now I would like to ask a few questions about you and your household.

Q5. What is your age? __ __ __

(years)

[ ] Refused =888

[ ] Don’t know =999

Q6. Are you Hispanic, Latino/a, or Spanish origin?

[ ] Yes =1

[ ] No =2

Q7. Which one of these groups would you say best represents your race?

*Please read:*

[ ] White =1

[ ] Black or African American =2

[ ] American Indian or Alaska Native =3

[ ] Asian =4

[ ] Pacific Islander =5

*Do not read:*

[ ] Other =6

[ ] No additional choices =8

[ ] Don’t know / Not sure =7

[ ] Refused =9

Q8. What is the highest grade or year of school you completed?

*Read only if necessary:*

[ ] Some grade school =1

[ ] High school =2

[ ] College graduate (2 or 4 year degree) =3

[ ] Post-graduate education =4

Q9. Are you currently…?

*Please read:*

[ ] Employed for wages =1

[ ] Self-employed =2

[ ] Out of work for 1 year or more =3

[ ] Out of work for less than 1 year =4

[ ] A Homemaker =5

[ ] A Student =6

[ ] Retired =7

[ ] Unable to work =8

*Do not read:*

[ ] Refused =9

Q10. Is your annual household income from all sources—

*Please read:*

[ ] Less than $25,000 =1

[ ] $25,000-$50,000 =2

[ ] $50,000-$75,000 =3

[ ] More than $75,000 *=4*

*Do not read:*

[ ] Don’t know =8

[ ] Refused =9

Q11. Do you own or rent your home?

[ ] Own =1

[ ] Rent =2

[ ] Other arrangement =3

*INTERVIEWER NOTE: “Other arrangement” may include group home, staying with friends or family without paying rent.*

*NOTE: Home is defined as the place where you live most of the time/the majority of the year.*

Q12. Indicate sex of respondent. *Ask only if necessary*.

[ ] Male =1

[ ] Female =2

Q13. How many persons in your household are

Male _ _

Female _ _

Q14. Was anyone in your household pregnant between January 9 and January 31, 2014?

[ ] Yes =1

[ ] No =2

[ ] Don’t know =8

[ ] Refused =9

Q15. How many people in your household are in each age category?

< 1 year _ _

1-17 years _ _

18-64 years _ _

65+ years _ _

**THE CHEMICAL SPILL**

The next set of questions asks about the chemical spill into the Elk River that occurred on January 9th, 2014.

Q16. When did someone in your household first learn about the chemical in the Elk River? *(Read choices)*

[ ] The same day as the spill (January 9, 2014) =1

[ ] The day after the spill (January 10, 2014) =2

[ ] Later than that =3

[ ] Don’t know =8

**If don’t know: GOTO Q18**

Q17. About what time of day did you first learn of the chemical in the Elk River?

_ _:_ _

*(military time; code 99:99 if don’t know/don’t remember)*

Q18. How did your household first learn about the chemical spill? (*Do not read; choose the one best response*)

[ ] Noticed odor in water =01

[ ] Noticed odor in the air =02

[ ] Phone call from water company =03

[ ] Word of mouth/talking to people =04

[ ] Television =05

[ ] land line call =06

[ ] cell phone call =07

[ ] Text message from friend/relative =08

[ ] Radio =09

[ ] Internet =10

[ ] Social media (e.g. Facebook, twitter) =11

[ ] Other =12

[ ] Don’t know/don’t remember = 88

**If other:** how? _______________________________

Q19. Please tell us sources of water that you had in your household at the time you first heard about the chemical spill. Did you have…

Q19a. [ ] Public water supply? =1

Q19b. [ ] Well water? =1

Q19c. [ ] Purchased water (e.g. bottled water)? =1

Q19d. [ ] Some other source? =1

**If Q19d (Other):** What was the source ? _________________________

**If not Q19c (no purchased water) GOTO COMMUNICATION.**

Q20. About how many gallons of purchased water did you have when you first heard of the chemical spill? Would you say you had…

[ ] Less than 1 gallon? =1

[ ] 1-2 gallons? =2

[ ] 3-5 gallons? =3

[ ] More than 5 gallons? =4

[ ] Don’t know =8

**COMMUNICATION QUESTIONS**

The next few questions ask about sources of information during the water contamination emergency. We’ll ask you to rate how clear, helpful, and trustworthy you felt information from each source was. Please rate each on a scale from A (excellent) to F (failed). You can also say you don’t know or don’t remember. First we’ll ask about information from federal health officials such as the Centers for Disease Control (CDC).

Q21. During the water emergency, how clear and understandable were information and announcements from federal health officials? Please rate from A to F *(write down letter grade or ‘X’ for don’t know/don’t remember) ___*

Q22. During the water emergency, how helpful was information and announcements from federal health officials? Please rate from A to F *(write down letter grade or ‘X’ for don’t know/don’t remember) ___*

Q23. During the water emergency, how trustworthy was information and announcements from federal health officials? Please rate from A to F *(write down letter grade or ‘X’ for don’t know/don’t remember) ___*

Now please think about information you may have received from state officials such as the Governor and state health and environmental directors.

Q24. During the water emergency, how clear and understandable were information and announcements from state officials? Please rate from A to F *(write down letter grade or ‘X’ for don’t know/don’t remember) ___*

Q25. During the water emergency, how helpful was information and announcements from state officials? Please rate from A to F *(write down letter grade or ‘X’ for don’t know/don’t remember) ___*

Q26. During the water emergency, how trustworthy was information and announcements from state officials? Please rate from A to F *(write down letter grade or ‘X’ for don’t know/don’t remember) ___*

Now please think about information you may have received from the West Virginia American Water Company.

Q27. During the water emergency, how clear and understandable were information and announcements from the water company? Please rate from A to F *(write down letter grade or ‘X’ for don’t know/don’t remember) ___*

Q28. During the water emergency, how helpful was information and announcements from the water company? Please rate from A to F *(write down letter grade or ‘X’ for don’t know/don’t remember) ___*

Q29. During the water emergency, how trustworthy was information and announcements from the water company? Please rate from A to F *(write down letter grade or ‘X’ for don’t know/don’t remember) ___*

Finally, please think about information you may have received from local officials such as the mayor, county commissioners, or the Kanawha Charleston Health Department.

Q30. During the water emergency, how clear and understandable were information and announcements from local officials? Please rate from A to F *(write down letter grade or ‘X’ for don’t know/don’t remember) ___*

Q31. During the water emergency, how helpful was information and announcements from local officials? Please rate from A to F *(write down letter grade or ‘X’ for don’t know/don’t remember) ___*

Q32. During the water emergency, how trustworthy was information and announcements from local officials? Please rate from A to F *(write down letter grade or ‘X’ for don’t know/don’t remember) ___*

The next questions ask about how your household used water during the emergency.

Q33. Did anyone in the household use the public water supply in your home at any point during the time that the ‘do not use’ order was in effect for your household (other than flushing the toilet or trying to put out a fire)?

[ ] Yes =1

[ ] No =2

[ ] Don’t know =9

**If not yes, GOTO Q35**

Q34. How was the water used? Did someone …*(repeat stem for prompt as needed)*

Q34a. [ ] Drink the water? =1

Q34b. [ ] Wash hands? =1

Q34c. [ ] Brush teeth? =1

Q34d. [ ] Cook with the water? =1

Q34e. [ ] Wash clothes? =1

Q34f. [ ] Shower or bathe in the water? =1

Q34g. [ ] Run the dishwasher or hand wash dishes? =1

Q34h. [ ] Give water to pets? =1

Q34i. [ ] Water plants? =1

Q34j. [ ] Use water some other way? =1

**If used some other way, how? _____________________________**

Q35. After the chemical spill, did anyone in your household try to get another source of water that was not part of the public water supply?

[ ] Yes =1

[ ] No =2

[ ] Don’t know =9

**If not Yes: GOTO ECONOMIC IMPACT**

Q36. What other water sources did your family use? *(Read choices)*

Q36a. [ ] Water distribution center in your town of residence =1

Q36b. [ ] Water distribution center outside of your town of residence =1

Q36c. [ ] Purchased from large store or grocery (e.g. Wal-Mart, Kroger) =1

Q36d. [ ] Purchased from nearby convenience store or gas station =1

Q36e. [ ] Well water on premises =1

Q36f. [ ] Water from a friend or relative =1

Q36g. [ ] Rainwater =1

Q36h. [ ] Another source =1

**If another source, what source?** ________________________________

**If did not use water distribution center (neither Q36a nor Q36b): GOTO Q38.**

Q37. How did you find out about the location of the water distribution center? *(Read choices)*

Q37a. [ ] TV =1

Q37b. [ ] Radio =1

Q37c. [ ] Word of mouth/talking to people =1

Q37d. [ ] Social media, for example Facebook or Twitter =1

Q37e. [ ] Other Internet source =1

Q37f. [ ] Newspaper =1

Q37g. [ ] Another source =1

**If another source, what source?** ________________________________

Q38. Did anyone in your household go without safe drinking water for one or more days?

[ ] Yes =1

[ ] No =2

[ ] Don’t know =9

**If not yes: GOTO Q40**

Q39. If your household was without safe drinking water for one or more days, what was the reason? *(Read choices)*

Q39a. [ ] Not enough money to purchase water =1

Q39b. [ ] No transportation =1

Q39c. [ ] Store was out of water =1

Q39d. [ ] Distribution site out of water =1

Q39e. [ ] Could not find distribution site =1

Q39f. [ ] Distribution site changed =1

Q37g. [ ] Distribution site closed =1

Q39h. [ ] Did not have clean containers =1

Q39i. [ ] Could not leave work =1

Q39j. [ ] Other reasons =1

**If other reasons, what were they?** _____________________________

Q40. Did anyone in your household need to travel outside of the chemical spill affected area to try to get water during the ‘do not use’ order?

[ ] Yes =1

[ ] No =2

**If no: GOTO Q42**

Q41. If you were able to get water outside the chemical spill area, was it*(read choices)*

Q41a. [ ] Purchased at a store =1

Q41b. [ ] From a friend or relative =1

Q41c. [ ] From another source =1

**If another source, what was it? _________________**

Q42. Did anyone in your household stay overnight for one or more days outside of your home in an effort to get safe water?

[ ] Yes =1

[ ] No =2

**If no: GOTO Economic Impact**

Q43. Did anyone in your household pay money to stay somewhere, such as a hotel or campsite?

[ ] Yes =1

[ ] No =2

**ECONOMIC IMPACT**

The next set of questions asks about how much the spill might have cost your household.

Q44. Did the water contamination crisis cause you to miss any days from work?

[ ] Yes =1

[ ] No =2

**If no: GO TO Q46**

Q45. How many days did you miss? _________________________

Q46. Was work performance limited due to a water-related illness or distress?

[ ] Yes =1

[ ] No =2

Q47. What is your profession?____________________________

Q48. Does anyone in the household own a business?

[ ] Yes =1

[ ] No =2

**If no: GOTO Q50**

Q49. Was the business ordered to close as a result of the chemical spill?

[ ] Yes =1

[ ] No =2

[ ] Don’t know =9

Q50. Was any member of your household told not to come in to work because of the chemical spill?

[ ] Yes =1

[ ] No =2

**If no: GOTO Q52**

Q51. Did anyone who missed work due to the chemical spill get paid for it?

[ ] Yes =1

[ ] No =2

Q52. About how much extra did your household spend for water, childcare, food, and other costs related to this crisis? *(ask respondent for dollar amount, if can’t say read choices, e.g., do you think it was less than $100?)*

[ ] <$100 =1

[ ] $100-250 =2

[ ] $250-500 =3

[ ] >$500 =4

[ ] Don’t know/don’t remember =9

**HEALTH EFFECTS**

The next set of questions asks about the health impact of the chemical spill on the household.

Q53. After the chemical spill, did anyone in your household have any illness that he or she felt was related to the chemical spill?

[ ] Yes =1

[ ] No =2

[ ] Don’t know/don’t remember =9

**If Q53 not YES: GOTO** **PSYCHOLOGICAL DISTRESS**

Q54. What type of symptoms did you or your household members have? Did they have…*(Read choices, repeat stem as prompt as needed)*

Q54a. [ ] Nausea or vomiting =1

Q54b. [ ] Diarrhea, stomach pain or cramps =1

Q54c. [ ] Rash or skin irritation =1

Q54d. [ ] Headache or dizziness =1

Q54e. [ ] Eye irritation =1

Q54f. [ ] Respiratory symptoms such as cough or wheezing =1

Q54g. [ ] Other symptoms =1

**If other symptoms, what were they?** ____________________________

Q55. When did the symptoms start? *(read choices, multiple responses ok as different household members may have had different experiences)*

Q55a. [ ] Before the do not use order =1

Q55b. [ ] During the do not use order =1

Q55c. [ ] After the do not use order was lifted =1

Q56. Did you or a member of the household seek medical attention or advice because of these symptoms during the days after the chemical spill?

[ ] Yes =1

[ ] No =2

**If no: GOTO Q58**

Q57. Where did they go for medical attention or advice? Did they go to…*(Read choices, repeat stem as prompt as needed)*

Q57a. [ ] Primary care physician =1

Q57b. [ ] Urgent care =1

Q57c. [ ] Emergency room =1

Q57d. [ ] Admitted to the hospital =1

Q57e. [ ] Poison control center =1

Q57f. [ ] Internet source =1

Q57g. [ ] Other source(s) =1

**If other source(s), what were they?** ____________________________

Q58. Was there anyone in household who was ill with symptoms related to the water who did NOT seek medical attention or advice?

[ ] Yes =1

[ ] No =2

**If no: GOTO PSYCHOLOGIC DISTRESS**

Q59. Why didn’t they seek medical attention or advice? *(Read choices)*

Q59a. [ ] No insurance =1

Q59b. [ ] No transportation =1

Q59c. [ ] Concerned about the cost =1

Q59d. [ ] Did not feel ill enough to seek medical attention =1

Q59e. [ ] Other reason(s) =1

**If other reason(s), what were they?** ____________________________

**PSYCHOLOGIC DISTRESS QUESTIONS**

It has been almost 90 days since the start of the water emergency. The next few questions ask about how you felt during the time of the water emergency compared to now.

Please respond to each question using the following scale:

1. All of the time

2. Most of the time

3. Some of the time

4. None of the time

8. Don’t remember/don’t know *(Do not read)*

9. Refused *(Do not read)*

Q60. How much of the time did you feel worried during the 30 days after the water emergency began? Was it*…(repeat scale as needed for prompt) ___*

Q61. How much of the time have you felt worried during the last 30 days? *(prompt with scale as necessary) ___*

Q62. How much of the time did you feel stressed during the 30 days after the water emergency began? *(prompt with scale as necessary) ___*

Q63. How much of the time have you felt stressed during the last 30 days? *(prompt with scale as necessary) ___*

Q64. How much of the time did you feel angry during the 30 days after the water emergency began? *(prompt with scale as necessary) ___*

Q65. How much of the time have you felt angry during the last 30 days? *(prompt with scale as necessary)*

Q66. How much of the time did you feel depressed during the 30 days after the water emergency began? *(prompt with scale as necessary) ___*

Q67. How much of the time have you felt depressed during the last 30 days? *(prompt with scale as necessary) ___*

Q68. Did anything happen in your life such as a birth, an illness, or a death, that the water crisis made worse?

[ ] Yes =1

[ ] No =2

**If yes: please tell us what that was _________________________**

**AFTER THE EVENT**

*Scale for reference*

1. All of the time

2. Most of the time

3. Some of the time

4. None of the time

8. Don’t remember/don’t know *(Do not read)*

9. Refused *(Do not read)*

The last few questions ask about the impact of the chemical spill on your household after the ‘do not use order’ was lifted.

Q69. Is your household currently using water from the public water supply?

[ ] Yes =1

[ ] No =2

[ ] Refused =8

[ ] Don’t know =9

**If not yes: GOTO Q71**

Q70. How are you using the water? Do you use it to …*(repeat stem for prompt as needed)*

Q70a. [ ] Drink? =1

Q70b. [ ] Wash hands? =1

Q70c. [ ] Brush teeth? =1

Q70d. [ ] Cook? =1

Q70e. [ ] Wash clothes? =1

Q70f. [ ] Shower or bathe? =1

Q70g. [ ] Run the dishwasher or hand wash dishes? =1

Q70h. [ ] Give water to pets? =1

Q70i. [ ] Water plants? =1

**If no one drinks the water (Q70a is no): GOTO Q73**

Q71. Are some household members NOT drinking the water?

[ ] Yes =1

[ ] No =2

**If no: GOTO Q73**

Q72. Are any of the household members who aren’t drinking tap water…*(read choices)*

Q72a. [ ] Children? =1

Q72b. [ ] Pregnant women? =1

Q72c. [ ] Ill? =1

Q73. BEFORE the chemical spill, did you believe that the public water supply that your household received was safe?

[ ] Yes =1

[ ] No =2

[ ] Refused =8

[ ] Don’t know =9

Q74. AFTER the ‘do not use ban’ was lifted, do you believe that the public water supply that your household is receiving is safe?

[ ] Yes =1

[ ] No =2

[ ] Refused =8

[ ] Don’t know =9

Q75. Does your household have ongoing needs or concerns in relation to the water emergency? Or is there anything else you would like to share with us related to the chemical spill that we did not cover in the survey?

[ ] Yes =1

[ ] No =2

[ ] Refused =8

[ ] Don’t know =9

**If yes, what? _______________________________________________**

This concludes the survey. Thank you very much for your help. If you have further questions about the survey, please call (304) 348-6494. Watch your newspaper or television for news about results of the survey, or call that number in 30 days for information about survey findings. Thank you again. Good bye.
