# Supplementary material for: Self-Reported Household Impacts of Large-Scale Chemical Contamination of the Public Water Supply, Charleston, West Virginia, USA
Source: PLoS One. 2015 May 7;10(5):e0126744. doi: 10.1371/journal.pone.0126744 (PMC4423935; doi:10.1371/journal.pone.0126744)
Supplement: S2 Text — (DOCX) [file pone.0126744.s002.docx]

**Details of survey administration:**

We obtained three lists of random telephone numbers in Kanawha county consisting of almost 4,000 land line numbers and 2,000 cellular phone numbers; and conducted telephone interviews beginning April 3, 2014 and continuing through April 8, 2014.

The first list (Batch 1L) consisted of 1,998 land line numbers used in 2012 for a community assessment survey. The Kanawha Coalition for Community Health Improvement provided KCHD with this list, which was originally used in conducting household surveys for the Community Health Assessment in 2012. The Coalition purchased randomly selected landline telephone numbers for Kanawha County households from Survey Sampling International (Shelton, CT). The landline sample consisted of 4,000 numbers. After the Coalition screened the list for disconnects and businesses, the 1,998 numbers used in this survey remained.

The second and third lists each consisted of 1,000 landline and 1,000 cellular telephone numbers purchased from Survey Sampling International in April 2014. These were designated Batches 2 and 3.

For the first 24 hours, interviewers used Batch 1 only. We then began using Batch 2, and when it was nearly exhausted, began using Batch 3, starting with landlines and then beginning cell phone numbers. We did not complete Batch 3 by the end of the survey period. Numbers were used sequentially from the lists as provided.

We identified disconnected and non-working numbers and fax machines as we called. We attempted three calls to each working number. If we reached an answering machine, we left a message and attempted to call back later. We also called back busy numbers. If we reached a person, we asked whether it was a business or residence, and, if a residence, whether it was within Kanawha County. We initiated interviews on residential numbers within Kanawha County.

We considered a call “attempted” if we dialed a number at least once. It was “completed” if a person answered the telephone, whether or not eligible, willing, and able to participate in the survey. We interviewed the person who answered the telephone, if age 18 or older. The survey denominator consisted of those individuals who answered the telephone and were eligible to participate. Respondents were those who were able and willing to participate for whom survey forms were initiated and at least one question answered. The response rate was the proportion of eligible persons who agreed to participate.

We defined an attempt rate as the number of telephone numbers we dialed divided by the number available, and a contact rate as the proportion of working numbers we called that we eventually reached a respondent.

We eliminated duplicate numbers, resulting in a total of 5,948 telephone numbers, of which we attempted to call 3,377 (attempt rate 56.7%). After excluding 482 disconnected/non-working numbers, we completed calls to 1,246 telephones, for a contact rate of 43%. Answering machines (n=1,056) were the major reason for noncontact for those numbers we attempted to call. Excluding 412 ineligible individuals and 385 who refused left 498 respondents, for a response rate of 59.8% (calculated as the number of respondents divided by the number completed less the number ineligible).

Attempt rates varied by batch and were consistent with the order in which we placed calls. The Batch 1 rate was 16.7% and the Batch 3 cell phone rate was 27.7%. Contact rates were about 10% higher for landlines than cellular telephones.

Table (a): The distribution of telephone calls

| **Batch** | **Calls Attempted** | **Calls Completed** | **Ineligible** | **Refused** | **Respondents** | **Numbers Available** |
| --- | --- | --- | --- | --- | --- | --- |
| 1L | 325 | 139 | 49 | 34 | 56 | 1945 |
| 2C | 958 | 232 | 110 | 81 | 41 | 1000 |
| 2L | 928 | 468 | 98 | 148 | 222 | 974 |
| 3C | 277 | 57 | 29 | 8 | 20 | 1000 |
| 3L | 846 | 333 | 102 | 77 | 154 | 974 |
| 9L | 43 | 17 | 2 | 10 | 5 | 55 |
| Total | 3377 | 1246 | 390 | 358 | **498** | 5948 |

L = landline; C =cellular phone; 9L = numbers that were duplicated between batches 1L and 2L or 1L and 3L

Table (b): The attempt, completion & response rates by Batch

| **Batch** | **Attempt Rate** | **Contact Rate** | **Response Rate** |
| --- | --- | --- | --- |
| 1L | 16.7% | 48.1% | 60.9% |
| 2C | 95.8% | 34.0% | 30.8% |
| 2L | 95.3% | 52.8% | 58.7% |
| 3C | 27.7% | 30.6% | 58.8% |
| 3L | 86.9% | 41.0% | 66.1% |
| 9L | 78.2% | 43.6% | 35.7% |
| Overall | 56.8% | 43.0% | 56.3% |
