# Supplementary material for: Self-Reported Household Impacts of Large-Scale Chemical Contamination of the Public Water Supply, Charleston, West Virginia, USA
Source: PLoS One. 2015 May 7;10(5):e0126744. doi: 10.1371/journal.pone.0126744 (PMC4423935; doi:10.1371/journal.pone.0126744)
Supplement: S3 Text — (DOCX) [file pone.0126744.s003.docx]

**Comparison with the CDC/ WV DHHR CASPER survey**

The CDC’s CASPER survey was a door-to-door interview, unlike the KCHD survey, which was based on telephone interviews. Though the survey instrument used in the CASPER survey contains many questions similar or identical to those used in the KCHD survey, there was no collaboration/ sharing of survey instruments, and these two surveys were completely independent of each other. The KCHD instrument was designed with the intent of performing limited two-way and possibly multivariate analyses; the CASPER instrument appears to be intended for univariate analysis, albeit weighted to represent the population in the geographic area.

Household demographics

The demographic questions in the CASPER instrument, like the KCHD’s, were based on the Behavioral Risk Factor Surveillance System (BRFSS) questions. Both the KCHD and CASPER used non-BRFSS income questions. The CASPER tool was compatible with KCHD’s, but had more response levels. The CASPER tool did not record respondent demographics, but did collect household composition information.

Table(i) compares household age and sex composition from the two surveys. KCHD households included more older individuals and fewer working age adults than the CASPER survey.

Because CASPER used a “*check all that apply*” question for race of household members, compared with KCHD’s question on race of the respondent, the two items are not comparable. However, it appears that the vast majority of respondents and household members were white. CASPER sought the highest education level of anyone in the household, whereas KCHD asked for respondent education. This could explain the apparently higher levels of education reported in the former, e.g., 57% vs 37% college educated and 14% vs. 7% advanced degree. The CASPER survey may have reached a higher-income population than the KCHD; the inclusion of Putnam County (relatively affluent compared with Kanawha County) residences could have contributed to the difference. The two surveys had similar refusal rates (13% CASPER vs. 12% KCHD) and “don’t know” response rates (5% vs 4%) to the income item.

Communication

In both surveys, 81% of households learned of the spill the first day. About half of both groups first learned of it from television (CASPER, 54% vs. KCHD, 51%). Word of mouth was an equally common source in both surveys (14% vs. 16%). The CASPER survey asked respondents to choose the most reliable source of information by medium; the KCHD asked for assessment of authorities (federal, state, local, and water company) on three dimensions: clarity, helpfulness, and trustworthiness. CASPER asked the date respondents learned about the “do not use” order; when “don’t know” responses were excluded; frequencies appear similar to the date they learned of the spill.

Thirty-eight percent of CASPER households learned of water distribution centers from television compared with sixty percent of KCHD’s, a significant difference. Frequencies for other sources of information were similar.

Preparedness

About half of households in each survey had bottled water on hand at the start of the emergency (CASPER, 53% vs. KCHD, 48%), but only a minority had a significant amount. CASPER asked about a “three day supply for each person” while KCHD inquired about the quantity on hand. Given the EPA estimated water consumption per person of 2 liters per day, and a median 3 person household size, the KCHD survey suggests about 6% of responding households had the approximately 5 gallons needed. The CASPER estimate is somewhat higher at 15%, but there are many uncertainties about estimating quantities and requirements that make both rates suspect.

Alternative water sources

The vast majority of respondent households in both surveys tried to get other water sources besides WVAWC. Similar proportions used water from all sources except for friends and relatives, which households in the CASPER survey used twice as often (CASPER, 44% vs. KCHD, 23%).

A greater proportion of CASPER respondents said they’d traveled out of the affected area to get water than KCHD respondents (49% vs. 29%), but the question format differed between the surveys. If those who traveled but did not actually get water are excluded, the difference would be smaller. Since the KCHD questions were multiple response, while the CASPER item had exclusive responses, we cannot directly compare the response frequencies for where the respondents obtained water out of their town.

Household impact

Eighteen percent of CASPER households stayed overnight out of area to get safe water, compared with twelve percent of KCHD households. Roughly twice the proportion of CASPER respondents stayed with family or friends than KCHD respondents (15% vs 7%). A small minority of each group (3% vs. 5%) paid to stay overnight. Proportions of respondents owning businesses and businesses ordered to close in the emergency were too small to compare. About one fifth of each group had a household member asked to stay home from work during the emergency (CASPER 22%, KCHD 16%), but CASPER household members were more likely to have received pay (13% vs. 6%). The CASPER survey did not assess monetary economic impact.

Health

The CASPER report specifies that the health questions in this survey were not intended to evaluate the chemical spill-related health problems. On the other hand, the KCHD instrument was intended to assess psychological distress as well as the quality of communication from different sources.

More KCHD than CASPER households reported illness related to the water (32% KCHD vs 23% CASPER). Because of the small number of households reporting illness in CASPER, it is difficult to compare specific symptom frequencies. The most frequent symptoms in both groups were skin rash and irritation, which seem to have affected the majority of households reporting any illness. Gastrointestinal symptoms were less common in both groups. In each group, symptom onset was more likely during or after the “do not use” order than before it. Only a minority of persons in households with symptomatic individuals sought medical care or advice (the two questions differed slightly). One quarter of KCHD respondent households with symptoms sought medical advice, versus almost one half of CASPER households, but the number with symptoms was small in the latter group. Few CASPER households had persons with signs of serious mental illness; CASPER did not ask about psychological distress.

Water use

Twenty three percent of KCHD households, and thirty seven percent of CASPER households reported using WVAWC water during the “do not use” order. Of those using the water, 13% of CASPER respondent households reported someone drinking it, vs. 37% of KCHD households. The absolute proportion of households reporting that someone drank water during the emergency differed by only 1% (10% CASPER vs. 9% KCHD). In both groups, only about 1/3 of households said they were drinking the water 3 months after the event (KCHD 34%, CASPER 33%).

In both surveys, the percent of household respondents who thought the water was safe declined precipitously after the event. In the KCHD survey, the rate declined from 87% to 29%; in CASPER, 85% to 36%.

In general

For the most important questions, there is striking agreement between response frequencies, in spite of substantial differences in demographics.

**Table (i): Age and Sex Composition of Households**

| **Percentage of households with one or more…** | **CASPER** | **KCHD** |
| --- | --- | --- |
| Male | 84% | 79% |
| Female | 89% | 89% |
| Pregnant female | 6% | 2%^++^ |
| Persons aged 2-17* | 25% | 23% |
| Persons aged 18-64 | 77% | 65%^++^ |
| Persons aged 65+ | 36% | 49%^++^ |

* KCHD estimated from 0-1 and 2-18 year old groups

++ KCHD estimate is outside the 95% C.I. of CASPER estimate

**Table (ii): Water Sources During Emergency**

| **Sources of water during emergency** | **CASPER** | **KCHD** |
| --- | --- | --- |
| Large store | 73% | 77% |
| Convenience store/gas station | 18% | 16% |
| Distribution center, town of residence | 48% | 55% |
| Distribution center, another town | 26% | 21% |
| Friend/relative | 44% | 23% |
| Well water | 3% | 3% |
| Rainwater | 14% | 10% |
