# Supplementary material for: Self-Reported Household Impacts of Large-Scale Chemical Contamination of the Public Water Supply, Charleston, West Virginia, USA
Source: PLoS One. 2015 May 7;10(5):e0126744. doi: 10.1371/journal.pone.0126744 (PMC4423935; doi:10.1371/journal.pone.0126744)
Supplement: S1 Table — (DOCX) [file pone.0126744.s004.docx]

**Table S1: Age and gender distribution of the respondents**

| Age groups (years) | Males | | Females | | Total | |
| --- | --- | --- | --- | --- | --- | --- |
|  | count | percent | count | percent | count | percent |
| 18-44 | 26 | 16.3% | 52 | 16.1% | 78 | 16.1% |
| 45-64 | 62 | 38.7% | 132 | 40.9% | 194 | 40.2% |
| ≥65 | 72 | 45.0% | 139 | 43.0% | 211 | 43.7% |
| Total | 160 | 100% | 323 | 100% | 483 | 100% |
