# Supplementary material for: Self-Reported Household Impacts of Large-Scale Chemical Contamination of the Public Water Supply, Charleston, West Virginia, USA
Source: PLoS One. 2015 May 7;10(5):e0126744. doi: 10.1371/journal.pone.0126744 (PMC4423935; doi:10.1371/journal.pone.0126744)
Supplement: S1 Fig — (DOCX) [file pone.0126744.s005.docx]

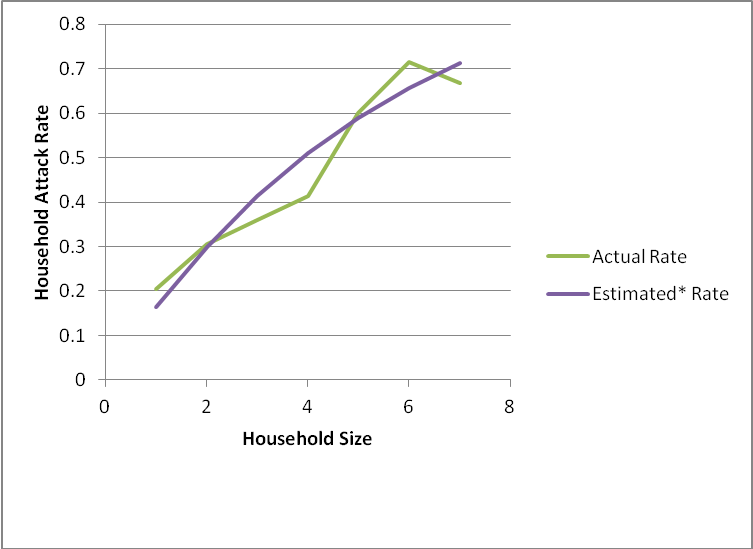


**We calculated the individual attack rate by minimizing the square difference between the actual household illness rate and the estimated rate based on the binomial distribution, assuming that cases of illness were independent of one another within households. The estimated rates did not differ significantly from the observed rates (p=0.993).*

**Fig. S1: Actual and estimated physical illness rates by household size**
