# Supplementary material for: Self-Reported Household Impacts of Large-Scale Chemical Contamination of the Public Water Supply, Charleston, West Virginia, USA
Source: PLoS One. 2015 May 7;10(5):e0126744. doi: 10.1371/journal.pone.0126744 (PMC4423935; doi:10.1371/journal.pone.0126744)
Supplement: S2 Fig — (DOCX) [file pone.0126744.s006.docx]

**
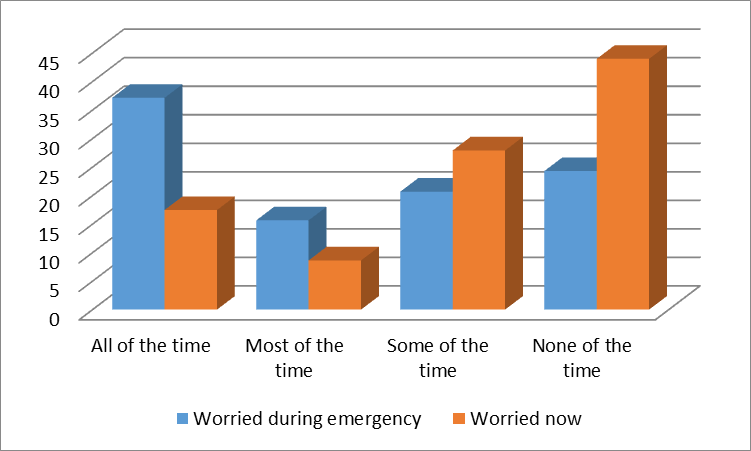
**

**Figure S2: Level of psychological distress during and after emergency, percent of respondents worried**
